# Assessment of the impact of the extension of vaccination mandates on vaccine coverage after 1 year, France, 2019

**DOI:** 10.2807/1560-7917.ES.2019.24.26.1900301

**Published:** 2019-06-27

**Authors:** Daniel Lévy-Bruhl, Laure Fonteneau, Sophie Vaux, Anne-Sophie Barret, Denise Antona, Isabelle Bonmarin, Didier Che, Sylvie Quelet, Bruno Coignard

**Affiliations:** 1 Santé publique France, Saint Maurice, France

**Keywords:** France, mandatory vaccination, vaccination coverage, vaccine confidence

## Abstract

One year after the extension of the childhood vaccination mandates to the 11 routine vaccinations for children under 2 years old, we estimated vaccination coverage through vaccine reimbursement data. Coverage for children born in 2018 has notably increased. Moreover, vaccine coverage for children and for vaccines not concerned by the law have also shown an increasing trend, supporting a positive impact of the ongoing communication strategy on vaccination, beyond the extension of vaccination mandates.

In December 2017, French parliamentarians passed a law extending the vaccination mandates for children from three (diphtheria, tetanus and poliomyelitis) to the 11 vaccinations included in the routine immunisation schedule of children under 2 years old. Children born from 1 January 2018 onwards are required to receive: three doses of a hexavalent vaccine which includes diphtheria, tetanus, poliomyelitis, pertussis, *Haemophilus influenza b* and hepatitis B antigens at age 2 and 4 months, with a booster dose at 11 months; three doses of the vaccine against invasive pneumococcal diseases with the same schedule; two doses of a vaccine against meningococcal C (MenC) diseases at age 5 and 12 months; and two doses of a vaccine against measles, mumps and rubella (MMR) at age 12 and 16–18 months [1].

The epidemiological, legal and societal determinants of such a decision have been described elsewhere [2]. Briefly, the main drivers of the decision were threefold: (i) the confusion created in many parents by the coexistence in the schedule of both mandatory and recommended vaccines, giving the false impression that the latter were less important or even optional [3]; (ii) the growing vaccine hesitancy in the French population, leading to insufficient vaccine coverage for most recommended vaccines [4]; and (iii) the translation of this insufficient coverage into an unacceptable burden of severe morbidity and mortality for some vaccine preventable diseases, including large outbreaks such as the measles epidemic observed in 2008–11 [5,6].

In practice, non-vaccinated children cannot be admitted to any kind of collective institutions such as nurseries, kindergarten, schools or any social activity if they have not complied with the vaccine mandates. No exemption other than medical contraindication is accepted. The law is not retroactive, meaning that only children born since 1 January 2018 are concerned [7].

This decision was highly debated and several experts expressed their concern about a potential counter-productive effect, fearing that it could ‘convert vaccine hesitancy into a more extreme anti-vaccination stance’ or ‘fuel further unfounded resistance to life-saving vaccines’ [8,9].

One year later, we present a first assessment of the impact of the law on vaccination coverage (VC) for children born in 2018 and therefore concerned by the measure. We also present data for some vaccinations given to children born before 2018 in order to assess the potential consequences of this change on VC of recommended vaccines.

## Source of vaccination coverage data

We used the National Social Security Reimbursement Database, which contains the reimbursement data for all drugs, including vaccines, for more than 99% of the population. Past experience has validated the use of this database to estimate VC through comparison with routine VC estimates obtained by the analysis of the child health certificates mandatorily filled at 24 months [10]. Virtually 100% of reimbursements of vaccines delivered in a given month are available two months later in the database. Data were extracted in March 2019, therefore allowing measurement of vaccination activities for 2018 as a whole.

## Vaccination coverage for children concerned by the vaccination mandates

Vaccine coverage for diphtheria, tetanus, poliomyelitis, pertussis and *Haemophilus influenzae b*, as measured by the 24 months child health certificates, has been at least 98% for many years because of the quasi-exclusive use of hexavalent or pentavalent (excluding the hepatitis B component) vaccines for primo vaccination. Estimates of coverage for these antigens cannot be generated through the National Social Security Reimbursement Database because we excluded from the analysis all children in their first year of life with no reimbursement of any DTP-containing vaccine. This was to account for the very low percentage of children (estimated ca 5%) who benefit from free vaccination in the Maternal and Child Health clinics [11]. To estimate the coverage for hepatitis B, we computed the proportion of children vaccinated with a hexavalent vaccine with, as a denominator, the number of children receiving either a pentavalent or an hexavalent vaccine and multiplied this figure by the proportion of children receiving a DTP-containing vaccine, obtained by the analysis of the 24 months health certificates (99%), to account for children who do not receive any vaccine. We compared vaccine coverage between children born January to May 2018 and January to May 2017.

For those same two cohorts of children, we compared vaccine coverage at 7 months of age for at least one dose of pneumococcal vaccine and the first dose of meningococcal C vaccine.

## Vaccination coverage for children not concerned by the vaccination mandates and for human papillomavirus (HPV) vaccine

We compared the VC for the first dose of MMR and the second dose of MenC vaccination at the age of 14 months, between children having reached their first birthday in 2018 and aged at least 14 months (children born between January and October 2017) and children born 1 year earlier (between January and October 2016). We also evaluated the number of human papillomavirus (HPV) vaccine doses reimbursed in 2018 for adolescent girls and compared this figure with similar ones for the years 2015–2017.

## Vaccination coverage comparisons

**Figure 1:**
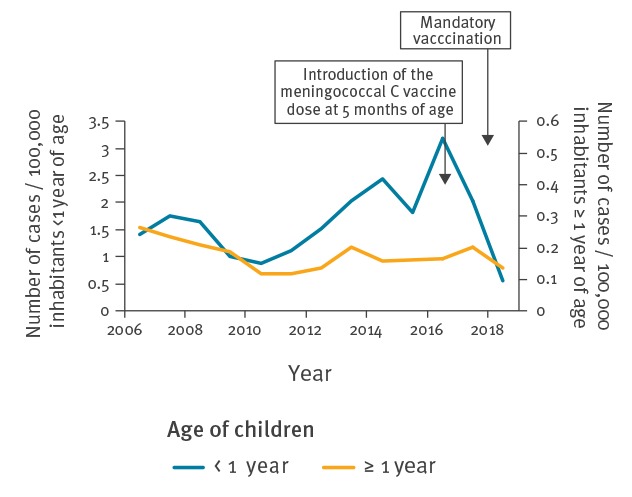
Incidence of invasive meningococcal infections according to age, France, 2006–2018

The proportion of infants, children under 1 year old, receiving a hexavalent vaccine increased from 93.1% in 2017 to 98.6% in 2018, corresponding to an increase of VC against hepatitis B from around 92% in 2017 to 98% in 2018. VC for at least one dose of pneumococcal vaccine increased from 98.0% to 99.4%, and vaccine coverage for the first dose of meningococcal C vaccine increased from 39.3% to 75.7% (Table 1). This sharp increase in MenC VC translated into a dramatic decrease in the number of invasive MenC disease cases notified in infants through the mandatory notification system, from 17 cases on average during the 2012–16 period to four in 2018, all in non-vaccinated individuals. This contrasts with the very limited decrease in incidence in individuals above 1 year of age in 2018 (Figure 1).

**Table 1:** Impact of vaccination mandates on vaccination coverage of children under 1 year old born January–May 2018, France

Vaccine	Vaccination coverage		
	Birth cohort		Gain in coverage (percent point)
	Infants born in January–May 2017	Infants born in January–May 2018	
Hepatitis B, at least 1 dose	92%	98%	**6%**
Pneumococcal, at least 1 dose	98.0%	99.4%	**1.4%**
Meningococcal C, first dose	39.3%	75.7%	**36.4%**

The increase in MMR first dose and MenC second dose VC between 2017 and 2018 was 3.0% and 5.7%, respectively. This compared with a 0.3% and 3.6% increase between 2016 and 2017 respectively (Table 2).

**Table 2:** Evolution of vaccination coverage at 14 months of age for vaccines scheduled at 12 months, France, 2016–2018

Vaccine	Vaccine coverage				
	Age reached			Gain in coverage 2016–17 (percent point)	Gain in coverage 2017–18 (percent point)
	12 months in 2016	12 months in 2017	12 months in 2018		
MMR, first dose	74.3%	74.7%	77.7%	0.3%	**3.0%**
Meningococcal C, second dose	55.8%	59.3%	65.0%	3.6%	**5.7%**

MMR: measles, mumps and rubella.

**Figure 2:**
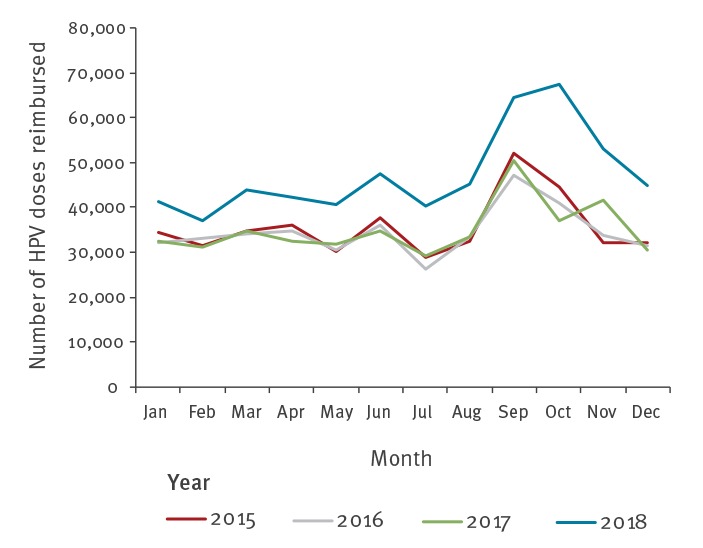
Number of human papillomavirus (HPV) vaccines reimbursed by month, France, 2015–2018

The number of doses of HPV vaccines reimbursed show a sharp increase between 2017 and 2018, contrasting with the almost stable volumes during the 2015–2017 period (Figure 2).

## Discussion

This first assessment of the impact of the extension of vaccination mandates on vaccination coverage is encouraging. It shows an increase in VC of infants concerned by the extension of the vaccination mandates. VC for the first dose of MenC will most likely continue to increase as time passes, when those children will be registered in a community requiring the completion of the schedule. More remarkable is the increasing trend seen for VC of children too old to have been concerned by the mandates. This suggests that the new law, at least at this stage, had no detrimental effect on vaccine coverage for vaccinations not yet concerned by the mandates or which remain recommended. This is especially true as VC measured at 14 months for the first dose of MMR and the second dose of MenC vaccination, for the sake of the current analysis, underestimate the future VC at 24 months for those children because of the usual catch-up during the second year of life. For the 2015 birth cohort, MMR first dose VC was estimated at 74.3% at 14 months and at 89.6% at 24 months through the health certificates (Table 2) [12]. We also observed a higher increase between 2018 and 2017 in the coverage for the second dose of MMR vaccination in children who reached their second birthday in the second semester of the year (from 75.5% to 78.4%) as compared with the increase in similar cohorts of children between 2016 and 2017 (from 74.0% to 75.5%)

The measles resurgence which started end of 2017 may have contributed to the increase in MMR VC. However, the increasing trend in vaccine coverage for children and vaccinations not concerned by the new mandates is likely to reflect, at least in part, the commitment of the French government in favour of vaccination at a high level, publicly expressed on several occasions by the Minister of Health and the Prime Minister, as well as the implementation, since 2017, by Santé publique France and its partners, of different actions aiming at promoting vaccination and countering vaccine hesitancy. One of the main achievements was the launching of a governmental website dedicated to vaccination (www.vaccination-info-service.fr) during the 2017 European Immunization Week. This site provides answers to most of the general public’s questions on vaccines and vaccinations. It has already received more than 6 million consultations. On the occasion of the 2019 European Immunization Week, an additional module of this website, one dedicated to healthcare professionals, was launched. It provides more insights into the various aspects of the National Immunisation Program, safety and effectiveness data, and on the evidence-base supporting the current recommendations.

The results of two surveys based on the same methodology conducted by the Vaccine Confidence Project in 2015 and 2018 were used to assess the improvement in the positive perception of the general public regarding vaccination overall. They show a decreasing proportion of French participants who disagree with the affirmation that vaccines are safe (from 41% to 23.7%) and effective (from 17.3% to 12.5%) [13,14]. However, much remains to be done to control or eliminate vaccine preventable diseases. In particular, the observed increase in MMR VC in young children will have very little impact on the current measles resurgence, which is mainly driven by the immunity gap in young adults who escaped both vaccination and natural infection in childhood. Nevertheless, the current situation is providing a unique momentum to strengthen the current efforts of the various vaccination stakeholders to restore confidence in vaccination, with the ultimate goal to control or eliminate vaccine preventable diseases and to lift the mandates.
